# Targeted Next Generation Sequencing for malaria research in Africa: current status and outlook

**DOI:** 10.1186/s12936-019-2944-2

**Published:** 2019-09-23

**Authors:** Anita Ghansah, Edwin Kamau, Alfred Amambua-Ngwa, Deus S. Ishengoma, Oumou Maiga-Ascofare, Lucas Amenga-Etego, Awa Deme, William Yavo, Milijaona Randrianarivelojosia, Plasmodium Diversity Network Africa, Lynette Isabella Ochola-Oyier, Gideon Kofi Helegbe, Jeffery Bailey, Michael Alifrangis, Abdoulaye Djimde

**Affiliations:** 1 Noguchi Memorial Institute for Medical Research, College of Health Sciences, University of Ghana, P. O. Box LG 581, Legon, Ghana; 2 Department of Emerging and Infectious Diseases (DEID), United States Army Medical Research Directorate –Africa (USAMR D-A), Kenya Medical Research Institute (KEMRI), Kisumu, Kenya; 3 U.S. Military HIV Research Program, Walter Reed Army Institute of Research, Silver Spring, MD USA; 4 Parasite Molecular Biology, Disease Control and Elimination, Medical Research Council Unit The Gambia at LSHTM, Atlantic Road Fajara, Banjul, The Gambia; 5 National Institute for Medical Research (NIMR), Tanga, Tanzania; 6 Bernhard Nocht Institute for Topical Medicine (BNITM), Hamburg, Germany; 7 West Africa Centre for Cell Biology of Infectious Pathogens, College of Basic and Applied Sciences, University of Ghana, Legon, Ghana; 8 Department of Parasitology, Faculty of Medicine and Pharmacy, Cheikh Anta Diop University, Dakar, Senegal; 9 Faculty of Pharmacy, Department of Parasitology and Mycology, Fe ´lix Houphoue ¨t-Boigny University, BPV 34, Abidjan, Côte d’Ivoire; 10 Malaria Research and Control Centre, National Institute of Public Health, BPV 47, Abidjan, Côte d’Ivoire; 11 Malaria Research Unit, Institut Pasteur de Madagascar, Avaradoha, BP 1274, 101 Antananarivo, Madagascar; 12 KEMRI-Wellcome Trust Collaborative Programme, P.O. Box 230, Kilifi, 80108 Kenya; 13 Department of Biochemistry and Molecular Medicine, School of Medicine and Health Sciences, University for Development Studies, P. O. Box TL1883, Tamale, Northern Region Ghana; 14 Warren Alpert Medical School, Brown University, 55 Claverick St, Rm 314B, Providence, RI 02903 USA; 15 Centre for Medical Parasitology, Department of Immunology and Microbiology, Faculty of Health and Medical Sciences, University of Copenhagen, Copenhagen, Denmark; 16 Malaria Research and Training Centre, University of Science, Techniques and Technologies of Bamako, Bamako, Mali; 17 Wellcome Trust Sanger Institute, Hinxton, UK

**Keywords:** Targeted Next Generation Sequencing, Malaria, Africa

## Abstract

Targeted Next Generation Sequencing (TNGS) is an efficient and economical Next Generation Sequencing (NGS) platform and the preferred choice when specific genomic regions are of interest. So far, only institutions located in middle and high-income countries have developed and implemented the technology, however, the efficiency and cost savings, as opposed to more traditional sequencing methodologies (e.g. Sanger sequencing) make the approach potentially well suited for resource-constrained regions as well. In April 2018, scientists from the Plasmodium Diversity Network Africa (PDNA) and collaborators met during the 7th Pan African Multilateral Initiative of Malaria (MIM) conference held in Dakar, Senegal to explore the feasibility of applying TNGS to genetic studies and malaria surveillance in Africa. The group of scientists reviewed the current experience with TNGS platforms in sub-Saharan Africa (SSA) and identified potential roles the technology might play to accelerate malaria research, scientific discoveries and improved public health in SSA. Research funding, infrastructure and human resources were highlighted as challenges that will have to be mitigated to enable African scientists to drive the implementation of TNGS in SSA. Current roles of important stakeholders and strategies to strengthen existing networks to effectively harness this powerful technology for malaria research of public health importance were discussed.

## Background

The evolution of next generation sequencing (NGS) technologies and the accompanying bioinformatic tools necessary to interpret the large quantity of sequence data have expanded the frontiers of biomedical research. These advances have impacted malaria research where these technologies are increasingly being performed for studying parasite diversity and antimalarial resistance in SSA [1–4], as discussed in the other papers in this series. Notably, NGS applications, such as Targeted Next Generation Sequencing (TNGS), which focuses on sequencing specific regions of the genome, tend to increase the sample load, the processing speed and at the same time, lower the costs of molecular analysis. Just as the traditional PCR technology was gradually introduced to every institution in SSA in the late 1990s, the relatively lower cost and speed of TNGS makes this technology the next natural step to be integrated into malaria research in SSA.

Currently, most malaria studies that have applied TNGS were funded by foreign agencies, spearheaded by collaborating non-SSA principal investigators, in collaboration with African scientists. The samples were processed for sequencing outside SSA after sample collection. Moreover, most of the TNGS data were generated and analysed in the non-SSA countries. In order to address the gap in TNGS data generation and analysis in Africa, the Plasmodium Diversity Network Africa (PDNA) [5] and research collaborators from non-SSA countries organized a workshop on the 18th of April 2018, as part of the MIM conference. The workshop was entitled “Targeted Next Generation Sequencing for Public Health” and hosted by the Institut de Recherche en Santé, de Surveillance Epidémiologique et de Formation, (IRESSEF), Diamniadio, Senegal. The workshop served as a platform for African researchers and their collaborating partners to discuss the status of TNGS applications in Africa, highlight opportunities and challenges and to discuss ways of bridging the gaps in application of TNGS. It was acknowledged that to successfully integrate TNGS technology for malaria research in SSA, several critical requirements are needed. These include; generating the key research/public health questions, funding, infrastructure and human resource development, engagement of policy makers in scientific deliberations, strengthening and harmonizing collaborations, and establishing/implementing a regulatory framework for operating NGS and TNGS in SSA.

## Highlights from the symposium

### Malaria targeted next generation sequencing and public health needs in Africa

A growing role of public health practitioners is to develop interventions for combating malaria and to evaluate the ability of these interventions to reduce morbidity and mortality in these populations. Several applications of TNGS for addressing the impact of such interventions were highlighted during the workshop. Table 1 outlines potential applications of TNGS in addressing important public health concerns in Africa, focusing on malaria. At the workshop, additional possibilities of personalized genomics and pharmacogenomics, and links between parasite genetics/drug resistance/host immune response and vaccine efficacy were also discussed.

**Table 1:** Potential role of targeted next generation sequencing in addressing malaria research of public health importance in Africa

Scientific objective	Application
Determination of the frequency and geographic distribution of genetic markers of antimalarial drug resistance	Early detection of drug resistance emergence and surveillance of the geospatial changes of antimalarial drug resistance
Diversity, frequency and geographic distribution of genetic differences in potential vaccine antigens among malaria parasites,	Support for antigen diversity studies in vaccine development and prediction of vaccine efficacy
Distribution and quantitative level of parasite DNA in human populations	Estimation of prevalence of submicroscopic infection
Determination of efficacy of diagnostic tools	Surveillance for geospatial distribution of parasites carrying hrp2/3 deletion Determination of RDT level of sensitivity
Detection of full range of parasite genotypes in a patient isolate	Increased sensitivity to determine whether parasites recurring after drug treatment are new infections or recrudescence/relapse of initial infections
Detection of very low levels of parasite DNA in patient samples	Improved detection of parasite prevalence even at low parasitaemia Improved quantitation of delayed parasite clearance

As discussed in other papers in this series, TNGS (and molecular tools in general) have significant potential, but have seen limited impact on public health in African countries. Although African scientists have spearheaded collecting and processing samples and in cases led the analysis of sequencing data [2, 4, 6–8], in order to fully operationalize TNGS to have its greatest impact for public health in Africa, projects need to be led and completed by African scientists in African institutions. By conducting this work in Africa, data can be generated in a timelier fashion, capacity built for research and data ownership issues minimized. The discrepancy between sample collection and driving studies is driven by a blend of general challenges facing researchers in SSA, such as funding, and more specific challenges relating to the implementation of TNGS in Africa.

## Challenges faced by sub-Saharan African scientists in the implementation of TNGS and the way forward

### Infrastructural development and cost

Despite the significant decrease in sequencing costs over the last decade, the prices of most of the sequencing instruments and the cost of establishing a sequencing centre remain very high and beyond the budget of most African institutions. The few African institutions that have acquired lllumina MiSeq sequencers are at various stages of operationalizing them, ranging from setting up of the sequencing centre to quality control testing; very few have begun to generate data. A large majority of African scientists obtain partially analysed sequence data from their collaborators and in rare cases outsource directly to commercial sequencing companies or through local agents of these companies. Numerous reasons were outlined during the discussion at the workshop, and it became quickly clear that logistical challenges are high on the list. For example, it is estimated that the establishment of an NGS facility costs USD 100,000–700,000 for the sequencing instruments alone, depending on the actual platform to be established [9]. In SSA, this cost may increase, depending on how the equipment is purchased. Equipment purchased through collaboration may come at reduced cost discounted through the collaborator, but with loss of warranty and maintenance agreements. On the other hand, those purchased through manufacturer’s sole agent in Africa are loaded with intermediary costs and profits, expensive shipment and customs costs. Similarly, the cost and difficulty of obtaining required reagents is a major barrier; it was mentioned during the discussions by several delegates that when cost comparisons between SSA, with purchases being done through intermediate African or Middle Eastern vendors, and European/North American institutions, costs were threefold higher in most African sites. Furthermore, often long clearance time at customs in SSA countries and as well, sudden changes of procedures at customs all add additional time and cost to the purchase. These costs barrier put together suggest that setting up a working sequencing platform is currently not affordable for most educational, research and clinical laboratories in developing countries.

To address some of the issues raised, the discussions centred on practical solutions. A first step might be the establishment of regional/sub-regional centres of excellence with central NGS facilities that serve a network of institutions/sub-regions. Such centres should be maintained jointly, providing full access to the facilities and hands on training for researchers from joint institutions, enabling collective sample processing (also reducing costs) to address relevant research questions of public health interest (e.g. Table 1). These sub-regional facilities would be equipped with additional NGS platforms as the technology evolves.

Centres of excellence are already being established to provide high-quality sequencing services for several laboratories and research groups. For example, the African Centre of Excellence for Genomics of Infectious Diseases (ACEGID) at Redeemer’s University (Nigeria) has been established with support from the World Bank and the US National Institute of Health (NIH) to serve several institutions in the surrounding region, including Senegal, Nigeria, and Sierra Leone [10]. The Africa Centre for Disease Control (CDC) is also engaging in the establishment of centres of excellence to track outbreaks and monitor re-emerging infectious diseases such as Lassa fever, and other viral haemorrhagic fevers, and Tuberculosis [11]. In West Africa, The Medical Research Council Unit The Gambia (MRCG-LSHTM) has purposed-built genomics and high-performance computing platforms accessible to West African networks, such as the PDNA. Creating NGS laboratory networks has several advantages; with higher projected use, they could negotiate with manufacturers, shippers and maintenance providers to ensure timely procurements and reduction of costs of reagents, and servicing of equipment. The network can have a single service contract for servicing of equipment and enabling visiting engineers service equipment from the whole sub-region in a single trip.

### Data generation and handling

TNGS applications involve epidemiology and sequence data collection. In some cases, the data may carry metadata containing sensitive information such as personal data (name, gender, date of birth, race), medical history, and family history of diseases. Such information must be handled carefully with regulations to protect the privacy and maintain the anonymity of the source of the sample, within the standard guidelines for biomedical research ethics. These standards are set and well-regulated outside SSA. In SSA countries however, since TNGS is still rare, the regulatory frameworks that stipulate quality and proficiency standards, specimen shipment requirements, data protection, may not be available or not operationalized, if they do exist [12]. During deliberations, the need for harmonized SOPs and standardized forms/tools to be used across laboratories was highlighted. This includes strong data capture systems and well-trained staff, with clear guidelines for sample preparation prior to sequencing, quality management, data capture and data management. This needs to occur in the context of strong supervision by an experienced principle investigator and lab management team. Agreed minimum data formats, quality systems such as tables, pie-charts, and bar-charts will be standardized and adopted for both public health and research purposes. This standardized data quality systems is very important because, it will simplify data visualization and dissemination for both public health experts and research scientists. This will be supported by institutions, which will need the data.

It is clear there is a need for the establishment and implementation of the legal, ethical and regulatory framework to cover TNGS data collection, handling and sharing. For instance, after the initial data analysis and use (reports, publication, planning) by the research group, data should be deposited at centralized local and international repositories for access upon request and following set and agreed regulations between African partners. The data should be released as soon as it is made available and this decision on data release should be made by the data owners via data sharing agreements. This could be guided by guidelines for the ethical handling of samples for genomics studies, as stipulated by H3Africa [13].

### Human resources (training) and infrastructural development for TNGS in Africa

Genomic data manipulation and analysis tools are essential for effective use of TNGS data. Genomics has become an interdisciplinary field that requires knowledge of biology, chemistry, statistics, computer science and bioinformatics. Thus, institutions applying genomics research need personnel who are adequately trained to generate and explore data. This is most lacking in SSA countries. Several open source tools and codes are available for basic data analysis; however, these need advanced analytical skills to identify, customize and apply to questions and data different from what they were originally developed to do. In addition, many commercialized advanced tools are too expensive for institutions in the low-income countries or require complicated licensing procedures even for single users [14]. Thus the need for human resource development and building of the infrastructure for data analysis to ensure that the trained personnel will be ready to apply the acquired skills was discussed. The consensus was that, operationalizing TNGS in the African regional centres will require specific training in the following general approaches; introduction of basic principles in genomic technologies, laboratory methodologies and bioinformatics analysis of resulting data, establishing bioinformatics software capabilities, and exploring collaborative applications of the genomic capabilities in public health [14, 15]. It is important to note that once targeted sequencing is completed, a specific bioinformatics pipeline is required to properly turn the “raw sequencing reads” into actionable outputs as discussed in the bioinformatics tools in this series. Furthermore, running and maintenance of bioinformatics pipelines need coding skills in multiple popular computer languages, e.g. R, Unix and python.

In addition, genomic data download and manipulation require fast and stable Internet connections that are not always available in low income countries, especially in Africa [15, 16]. The group agreed that local control of data by those who plan and carry out the collection and appropriate preservation of samples is important. This can be efficiently accomplished if groups analyse their data locally, no matter where the sequences and initial bioinformatics pipelines were generated. This will require reliable high capacity Internet access: local hardware and human resources, for example, High Performance Computing (HPC) streams and human resources—molecular biologists, computational biologists, and bioinformaticians. Currently, SSA has a few bioinformaticians and software users (biologists) capable of conducting this work. Initiatives like the H3AbioNet are building capacity for bioinformatics in Africa, but mainly in human genetics. PDNA’s Wellcome Trust DELTAS grant, Developing Excellence in Genomics for Malaria Elimination (DELGEME) on the other hand is training enthusiastic young Africans in bioinformatics, with emphasis on malaria (parasite, vector and vertebrate host). Partnerships with these initiatives and our collaborators for training and development of pipelines and tools for high quality data analysis by all was discussed. These technical supports could be both online and physical. Training programs in bioinformatics, including post-graduate diplomas, internships/short courses, post-doctoral fellowships and mentorship programmes with contributions from all stakeholders are all important.

The group also sees a future that utilizes innovative modern sequencing technologies with minimal infrastructural requirements. Emerging genome sequencing technologies, such as MinION by Oxford Nanopore Technologies, which have minimized the requirements of adopting this NGS technology with affordable devices, preparation kits and standard computer or even portable devices (phone and tablets). Although the technology is still new and developing, it nonetheless represents a promising solution to a wide adoption of genomics across a range of genome sequencing applications [17].

### Research funding

Lack of funding for biomedical research in Africa, has had an adverse consequence on the scientific development of the continent [15, 18–21]. Although the past decade has seen an increase in funding from research support initiatives like The Human Heredity and Health in Africa (H3Africa) initiative of the NIH and the Wellcome Trust [19], the DELTAS, FLAIR and APTI initiatives of the African Academy of Science, funding from Africa CDC, WAHO, ECOWAS, EAC and CEMAC, most African scientist including those working on malaria remain largely underfunded. This is a major impediment to molecular research in general and malaria genomics. Current trends indicate successful grant funding mostly directed at networks or consortia rather than individual scientists. An example is the PDNA, which has been spearheading research and training to address malaria genomic diversity studies to support elimination. The workshop discussed the increase in governmental interest in some African countries and the need for more such support. A typical example can be found in South Africa where genomics research benefits from government-sponsored grants from the National Research Foundation and other agencies [22, 23]. Other African countries should emulate South Africa by allocating more public funds to support smaller TNGS studies in home countries.

## Strengthening of collaborations and publications

Genome science and technologies are transforming life sciences globally in many ways and becoming a highly desirable area for international collaboration to strengthen global health. Collaboration between African scientists and their northern partners in genomic research, such as TNGS for malaria research will undoubtedly result in a substantial increase in scientific capacity. Several initiatives have been set up to support such collaborations [24]. A recent example is the public health collaboration by The Genome Science Program at the LA National Laboratory, USA and research institutions in several developing countries including Jordan, Uganda, and Gabon [25]. PDNA and collaborators will provide support for establishing the centres of excellence through training. To ensure credit is given or assured for the many people who have contributed to the data generation and analysis pipeline: planning and execution of the original study, molecular analysis, bioinformatics to produce initial output, data management and analysis of the output in local context, data archive and use or reuse. All those who have worked on the project will be listed in any publications with their specific contribution. A Digital Object Identifier (DOI) will be assigned to each dataset and will be listed so that universities and research Institutions can recognize data generators when the DOI is quoted in a publication. Appropriate locations for permanently archiving data are growing. For example, a Nature journal on archived data, facilitates uploading/archiving open source data and the accompanying metadata, and the group supported this approach to data reuse.

## Summary and conclusions

The revolution in the sequencing world is opening new frontiers for biomedical research and Africa must benefit from application of TNGS for malaria research. To achieve this, a number of challenges must be addressed. Firstly, there is the need for strategies to overcome financial and logistical challenges in establishing and maintaining infrastructure. This includes long-term support for scientist and infrastructure (stable environmental temperature, stable electrical power supply, reliable and efficient Internet access). Stable power and internet are becoming more accessible across Africa, but heavily biased toward urban agglomerates away from the centres of malaria transmission. Secondly, most African malaria research scientists and technicians are experienced biologists, but lack bioinformatics and quantitative/numeric expertise. Training a biologist in the techniques of genome science and data generation is relatively straightforward, but achieving informatics proficiency is a major hurdle, given very basic computational knowledge. Thirdly, funding remains a major challenge to research and training to boost NGS adoption and translation across most biomedical research themes in Africa. Development of regional or sub-regional centres of excellence will allow for reduced financial burden on scientists and facilitate collaboration. Centres will be collaborative hubs that will support molecular surveillance with high temporal, geographic, and information resolution to support local public health authorities in identifying and monitoring important phenotypes such as drug resistance. A network of African TNGS centres that can rapidly provide high-resolution genomic data that will help improve the speed and accuracy of detection and monitoring, and reduce the global threat from malaria drug resistance is urgently required.

Successful scientific partnerships and sustainable technical capacity are essential for establishing TNGS for malaria research in Africa. More local governmental support and funding for malaria TNGS research will be required. Scientists must engage the policy makers more effectively in research activities. This will require a carefully crafted regulatory framework for community engagement in genomics, data collection and storage, and regionally coordinated TNGS wet laboratory procedures, data ownership, management and analysis. Sustainability of the TNGS science capabilities with partners is a key concern.

There is the need for a continued improvement of local expertise through training. Sustainable use of the established TNGS technologies will be strengthened by performing regular research projects. Over time, this approach will enable African scientists to gain the expertise to develop genomic capabilities and continue on a path to sustainability. The question, however, is who should be directing these decisions and how? Since PDNA is leading in these discussions, the network will continue to drive the questions to be addressed with the TNGS platform and support the training of African scientist in bioinformatics.

## Data Availability

Not applicable

## Abbreviations

NGS: Next Generation Sequencing
TNGS: Targeted Next Generation Sequencing
SSA: sub-Saharan Africa
PDNA: Plasmodium Diversity Network Africa
DOI: Digital Object Identifier
NIH: National Institute of Health
